# A novel prediction tool for mortality in patients with acute lower gastrointestinal bleeding requiring emergency hospitalization: a large multicenter study

**DOI:** 10.1038/s41598-024-55889-7

**Published:** 2024-03-04

**Authors:** Naoyuki Tominaga, Eiji Sadashima, Tomonori Aoki, Minoru Fujita, Katsumasa Kobayashi, Atsushi Yamauchi, Atsuo Yamada, Jun Omori, Takashi Ikeya, Taiki Aoyama, Yoshinori Sato, Takaaki Kishino, Naoki Ishii, Tsunaki Sawada, Masaki Murata, Akinari Takao, Kazuhiro Mizukami, Ken Kinjo, Shunji Fujimori, Takahiro Uotani, Hiroki Sato, Sho Suzuki, Toshiaki Narasaka, Junnosuke Hayasaka, Tomohiro Funabiki, Yuzuru Kinjo, Akira Mizuki, Shu Kiyotoki, Tatsuya Mikami, Ryosuke Gushima, Hiroyuki Fujii, Yuta Fuyuno, Takuto Hikichi, Yosuke Toya, Kazuyuki Narimatsu, Noriaki Manabe, Koji Nagaike, Tetsu Kinjo, Yorinobu Sumida, Sadahiro Funakoshi, Kiyonori Kobayashi, Tamotsu Matsuhashi, Yuga Komaki, Kuniko Miki, Kazuhiro Watanabe, Mitsuru Kaise, Naoyoshi Nagata

**Affiliations:** 1 Department of Gastroenterology, Saga-Ken Medical Centre Koseikan, 400 Nakabaru, Kasemachi, Saga, 840-8571 Japan; 2 Department of Medical Research Institute, Saga-Ken Medical Centre Koseikan, Saga, Japan; 3 Department of Gastroenterology, Graduate School of Medicine, The University of Tokyo, Tokyo, Japan; 4 Division of Endoscopy and Ultrasonography, Department of Clinical Pathology and Laboratory Medicine, Kawasaki Medical School General Medical Center, Okayama, Japan; 5 Department of Gastroenterology, Tokyo Metropolitan Bokutoh Hospital, Tokyo, Japan; 6 Department of Gastroenterology and Hepatology, Kitano Hospital, Tazuke Kofukai Medical Research Institute, Osaka, Japan; 7 Department of Gastroenterology, Nippon Medical School, Graduate School of Medicine, Tokyo, Japan; 8 Department of Gastroenterology, St. Luke’s International University, Tokyo, Japan; 9 Department of Gastroenterology, Hiroshima City Asa Citizens Hospital, Hiroshima, Japan; 10 Division of Gastroenterology and Hepatology, Department of Internal Medicine, St. Marianna University School of Medicine, Kanagawa, Japan; 11 Department of Gastroenterology and Hepatology, Center for Digestive and Liver Diseases, Nara City Hospital, Nara, Japan; 12 Department of Gastroenterology, Tokyo Shinagawa Hospital, Tokyo, Japan; 13 Department of Endoscopy, Nagoya University Hospital, Aichi, Japan; 14 Department of Gastroenterology, National Hospital Organization Kyoto Medical Center, Kyoto, Japan; 15 Department of Gastroenterology, Tokyo Metropolitan Cancer and Infectious Diseases Center Komagome Hospital, Tokyo, Japan; 16 Department of Gastroenterology, Oita University, Oita, Japan; 17 Department of Gastroenterology, Chikushi Hospital, Fukuoka University, Fukuoka, Japan; 18 Department of Gastroenterology, Nippon Medical School, Chiba Hokusoh Hospital, Chiba, Japan; 19 Department of Gastroenterology, Japanese Red Cross Shizuoka Hospital, Shizuoka, Japan; 20 Division of Gastroenterology, Graduate School of Medical and Dental Sciences, Niigata University, Niigata, Japan; 21 Department of Gastroenterology and Hepatology, Center for Digestive Disease and Division of Endoscopy, University of Miyazaki Hospital, Miyazaki, Japan; 22 Department of Gastroenterology, University of Tsukuba, Ibaraki, Japan; 23 Division of Endoscopic Center, University of Tsukuba Hospital, Ibaraki, Japan; 24 Department of Gastroenterology, Toranomon Hospital, Tokyo, Japan; 25 Emergency and Critical Care Center, Saiseikai Yokohamashi Tobu Hospital, Kanagawa, Japan; 26 Department of Emergency Medicine, Fujita Health University Hospital, Aichi, Japan; 27 Department of Gastroenterology, Naha City Hospital, Okinawa, Japan; 28 Department of Internal Medicine, Tokyo Saiseikai Central Hospital, Tokyo, Japan; 29 Department of Gastroenterology, Shuto General Hospital, Yamaguchi, Japan; 30 Division of Endoscopy, Hirosaki University Hospital, Aomori, Japan; 31 Department of Gastroenterology and Hepatology, Graduate School of Medical Sciences, Kumamoto University, Kumamoto, Japan; 32 Department of Gastroenterology and Hepatology, National Hospital Organization Fukuokahigashi Medical Center, Fukuoka, Japan; 33 Department of Medicine and Clinical Science, Graduate School of Medical Sciences, Kyushu University, Fukuoka, Japan; 34 Department of Endoscopy, Fukushima Medical University Hospital, Fukushima, Japan; 35 Division of Gastroenterology, Department of Internal Medicine, Iwate Medical University, Iwate, Japan; 36 Department of Internal Medicine, National Defense Medical College, Saitama, Japan; 37 Division of Endoscopy and Ultrasonography, Department of Clinical Pathology and Laboratory Medicine, Kawasaki Medical School, Okayama, Japan; 38 Department of Gastroenterology and Hepatology, Suita Municipal Hospital, Osaka, Japan; 39 Department of Endoscopy, University of the Ryukyus Hospital, Okinawa, Japan; 40 Department of Gastroenterology, National Hospital Organization Kyushu Medical Center, Fukuoka, Japan; 41 Department of Gastroenterological Endoscopy, Fukuoka University Hospital, Fukuoka, Japan; 42 Department of Gastroenterology, School of Medicine, Kitasato University, Kanagawa, Japan; 43 Department of Gastroenterology and Neurology, Akita University Graduate School of Medicine, Akita, Japan; 44 Digestive and Lifestyle Diseases, Kagoshima University Graduate School of Medical and Dental Sciences, Kagoshima, Japan; 45 Department of Gastroenterological Endoscopy, Tokyo Medical University, Tokyo, Japan; 46 Department of Gastroenterology and Hepatology, National Center for Global Health and Medicine, Tokyo, Japan

**Keywords:** Gastroenterology, Colonoscopy, Gastrointestinal diseases

## Abstract

The study aimed to identify prognostic factors for patients with acute lower gastrointestinal bleeding and to develop a high-accuracy prediction tool. The analysis included 8254 cases of acute hematochezia patients who were admitted urgently based on the judgment of emergency physicians or gastroenterology consultants (from the CODE BLUE J-study). Patients were randomly assigned to a derivation cohort and a validation cohort in a 2:1 ratio using a random number table. Assuming that factors present at the time of admission are involved in mortality within 30 days of admission, and adding management factors during hospitalization to the factors at the time of admission for mortality within 1 year, prognostic factors were established. Multivariate analysis was conducted, and scores were assigned to each factor using regression coefficients, summing these to measure the score. The newly created score (CACHEXIA score) became a tool capable of measuring both mortality within 30 days (ROC-AUC 0.93) and within 1 year (C-index, 0.88). The 1-year mortality rates for patients classified as low, medium, and high risk by the CACHEXIA score were 1.0%, 13.4%, and 54.3% respectively (all *P* < 0.001). After discharge, patients identified as high risk using our unique predictive score require ongoing observation.

## Introduction

Patients with acute lower gastrointestinal bleeding (ALGIB) present with symptoms of hematochezia and sometimes require emergency hospitalization^1–5^. ALGIB can occasionally become severe and potentially fatal^6–8^. Gastrointestinal bleeding is categorized into upper gastrointestinal bleeding (AUGIB) and ALGIB, but ALGIB should be investigated separately from AUGIB. This is because, unlike AUGIB, there is no drug treatment such as acid secretion inhibitors for ALGIB, and epidemiological studies are crucial. Identifying prognostic factors for ALGIB could aid in its management.

Previous studies assessing the risk of long-term mortality (≥ 1 year) following hospitalization for ALGIB have been limited to a few single-center studies with small sample sizes (n < 500)^9,10^, and several variables potentially influencing mortality risk, such as vital signs, general condition, comorbidities, and in-hospital management, have not been evaluated^2,10^.

Furthermore, there is a need for careful follow-up of high-risk ALGIB patients, but a predictive scoring system that stratifies long-term mortality risk and identifies high-risk patients has not yet been established. Predictive scores for short-term mortality (30-day mortality or in-hospital mortality) have been reported in some previous studies^5,7,11,12^. However, the reported short-term mortality rates vary widely from 0.6 to 10.9%, lacking reliability^7,11,12^. Moreover, a study using a database of over 6000 patients based on the International Classification of Diseases codes found that factors such as admission vital signs and in-hospital rebleeding, which are involved in bleeding, were not considered significant risk factors for mortality^11^.

To address these issues, we conceived this study, believing that larger-scale, multicenter collaborative research is necessary. Computerized tomography (CT) and endoscopy are valuable modalities for diagnosing ALGIB, identifying the source of bleeding, and guiding treatment^13–16^. However, in previously reported large-scale studies, these data were insufficient. Considering this, we utilized nationwide data based on high diagnostic rates with adequately collected CT and endoscopy data to assess both short-term and long-term mortality rates. Additionally, since factors present at the time of hospital admission are expected to strongly influence short-term mortality, an ideal predictive tool would be one that can forecast short-term mortality at the time of admission. For long-term mortality, it is anticipated that factors present at the time of admission, as well as in-hospital management, contribute to mortality, hence an ideal predictive tool would be one that can estimate future mortality rates at discharge. Taking these elements into account, we developed a practical scoring system to determine short-term and long-term mortality risks and to stratify patients with high risk of death.

## Methods

### Study design, setting, and participants

This retrospective, multicenter, observational study was conducted between January 2010 and December 2019. It included patients with acute hematochezia who presented in an ambulatory setting and were urgently admitted to 49 hospitals across Japan. The decision for emergency admission was made by emergency physicians or gastroenterology consultants. The patient information gathered is reported as part of the CODE BLUE J-study (Colonic DivErticular Bleeding Leaders Update Evidence from a Multicenter Japanese Study)^17,18^. This study was conducted in accordance with the principles of the Declaration of Helsinki. As a retrospective study, the central institution (Tokyo Medical University) approved the use of an opt-out method, thereby waiving the necessity to obtain informed consent from patients. The central institution (Tokyo Medical University) has established a licensing committee/institutional review board for approving research involving human subjects. The research protocol was approved by the Tokyo Medical University Institutional Ethics Committee (T20190244). In this study, a single review by this ethics committee was applied and approved across all institutions (Supplementary Table 1).

This study aims to accurately predict mortality using clinical indicators present at the onset of the disease. Patients with ALGIB commonly visit the emergency department with acute hematochezia as their main complaint. “Hematochezia” refers to the discharge of red blood from the anus, which is predominantly indicative of lower gastrointestinal bleeding, but can also include cases of upper gastrointestinal bleeding. However, in actual clinical settings, it is not always possible to immediately determine the source of bleeding upon patient arrival. Therefore, to create a clinically relevant score, this study utilized data from patients presenting with hematochezia.

**Figure 1:**
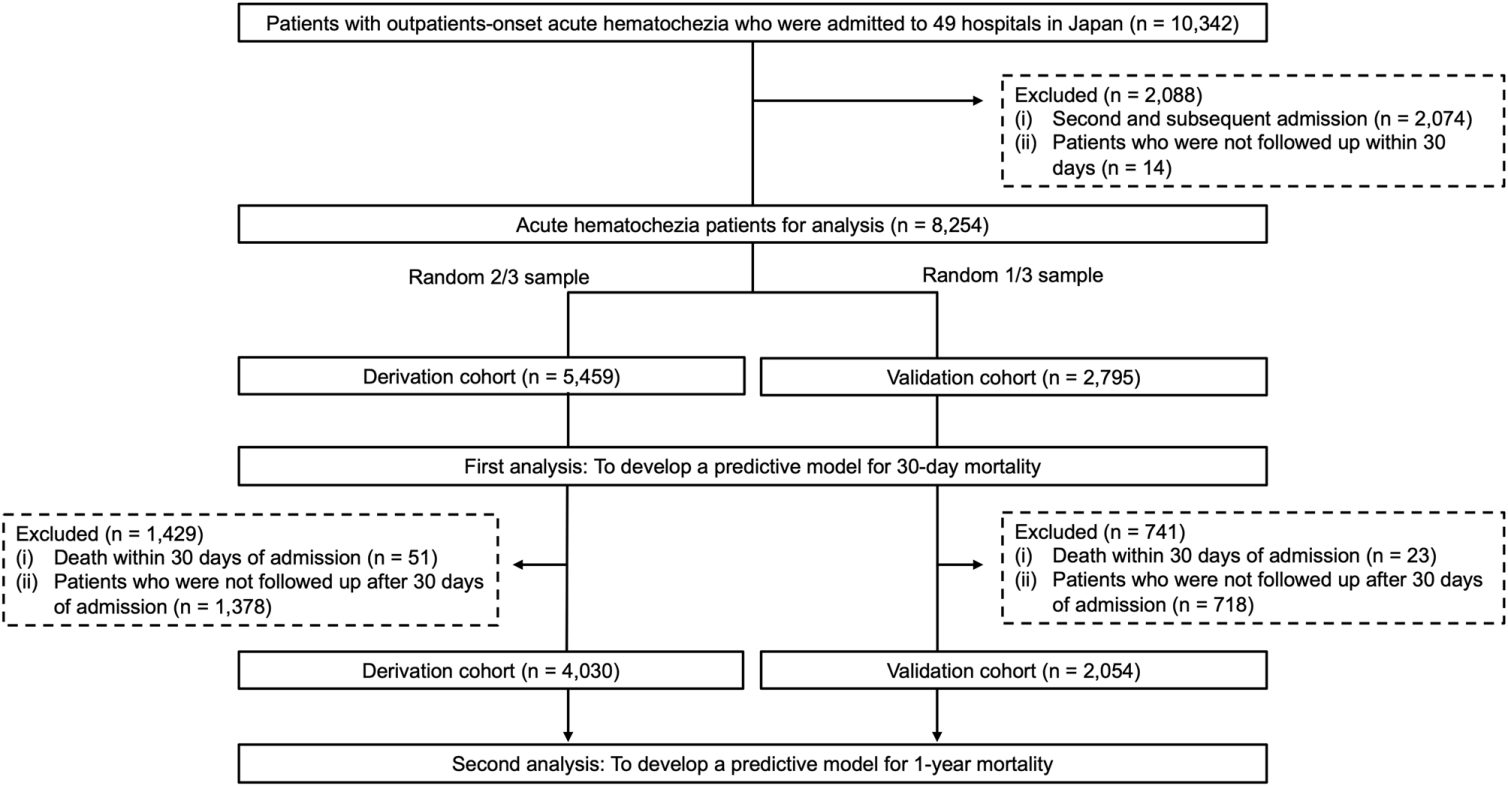
Flow chart.

The remaining 8254 patients were divided using a random number table into a 2:1 ratio for a validation group to assess reliability and a derivation cohort to create a new score. In this study, short-term mortality was defined as death within 30 days, and long-term mortality as death within 1 year. All patients were used for predicting mortality within 30 days, the first part of our study. For the second part of our study, patients who died within 30 days of admission (n = 74) and those who were not followed up after 30 days of hospitalization (n = 2096) were excluded. The remaining 6084 cases were used for predicting 1-year mortality (Fig. 1).

Among patients with acute hematochezia in the original cohort (n = 10,342), we excluded patients with second or subsequent admissions (n = 2074) and patients who were not followed up within 30 days (n = 14). The remaining 8254 patients were divided using a random number table into a 2:1 ratio for a validation group to assess reliability and a derivation cohort to create novel scores. In this study, short-term mortality was defined as death within 30 days, and long-term mortality as death within 1 year. All patients were used for predicting mortality within 30 days, the first part of our study. For the second part of our study, patients who died within 30 days of hospitalization (n = 74) and patients who were not followed up after 30 days of admission (n = 2096) were eliminated, and the remaining 6084 patients were used to forecast 1-year mortality (Fig. 1). Follow-up was defined as cases with at least one hospital visit and examination performed as needed. The examination was conducted on patients with acute hematochezia (including AUIGB) rather than ALGIB because the main goal of this study was to properly predict mortality using clinical indicators at the time of onset.

### Variables and outcomes

Data were collected from patient files and electronic endoscopic databases. In addition to in-hospital care data, baseline characteristics, including performance status (PS), vital signs, symptoms, initial laboratory findings, medications, and comorbidities, were collected. Comorbidities were collected to calculate the Charlson comorbidity index (CCI)^19^. In-hospital management included etiologies of bleeding sources (e.g., diverticular bleeding, colitis, ulcerative lesion, hemorrhoid, colorectal cancer, angioectasia, radiation proctitis, miscellaneous, and unknown origin) and in-hospital procedures (e.g., transfusion, endoscopic treatment, interventional radiology, and surgery). Endoscopic treatment was decided by the endoscopist following each hospital policy and included clipping, coagulation, band ligation, snare ligation, and hypertonic saline–epinephrine.

Mortality within 30 days of hospitalization was predicted using factors present at the time of admission, based on the assumption that these factors are strongly associated with death. For mortality within 1 year of hospitalization, it was assumed that in-hospital management factors also play a role in death, so these were added to the admission factors to predict mortality within one year. The list of predictive factors used is compiled in Supplementary Table 2. The cut-off values used were cited from papers on the prognosis and rebleeding of ALGIB and papers related to cachexia^2,5,20–22^. For the cut-off value of hematocrit, it was calculated using the cut-off value of hemoglobin used in this study and the normal mean corpuscular hemoglobin concentration.

Among the 6084 patients who were followed up for at least a year, the primary outcome was mortality within 1 year of admission, which was defined as death due to any reason after a month of admission until 365 days of admission. The secondary outcome was mortality within 30 days of admission, which was determined in the 8254 patients who were followed up for at least 30 days and who died for any reason within 30 days of admission; this period included the hospitalization period. Confirmation of death was determined from institutional medical records and death certificates, and death causes were categorized as hemorrhage-related or non-hemorrhage-related^23^. The cause of non-hemorrhage-related death was identified as a disease diagnosed by clinical examination, imaging studies, or autopsy.

### Statistical analyses

All patients were divided into the derivation and the validation cohorts at a 2:1 ratio using a random number table. The proportions between the two groups were compared using the Mann–Whitney U test or Fisher’s exact tests. The statistical significance level was P < 0.05. A complete-case analysis approach was employed, utilizing all non-missing observations available in the relevant analyses. Table 1 and Supplementary Table 3 include details about the missing data.

**Table 1:** Acute hematochezia patient characteristics in the validation and derivation cohorts.

Characteristics	Patients, no. (%)		*P* value*	Missing values
	Derivation cohort (n = 5459)	Validation cohort (n = 2795)		
Age ≥ 70 years	3232 (59.2)	1641 (58.7)	0.670	0
Sex (male)	3300 (60.5)	1673 (59.9)	0.601	0
BMI ≤ 18.5	651 (12.8)	328 (12.6)	0.773	185 (6.6)
Current drinker	2202 (40.3)	1103 (39.5)	0.723	441 (15.8)
Current smoker	910 (16.7)	447 (16.0)	0.774	387 (13.8)
Performance status ≥ 2	698 (12.8)	357 (12.8)	0.287	22 (0.8)
Hemodynamics				
Systolic blood pressure ≤ 100 mmHg	739 (13.5)	382 (13.7)	0.892	46 (1.6)
Heart rate ≥ 100/min	1087 (19.9)	553 (19.8)	0.884	54 (1.9)
Symptom				
Altered mental status	371 (6.8)	214 (7.7)	0.160	1 (0.0)
Abdominal pain	1004 (18.4)	489 (17.5)	0.319	4 (0.1)
Diarrhea	607 (11.1)	313 (11.2)	0.941	6 (0.2)
Laboratory data				
Hemoglobin < 12 g/dL	3157 (57.8)	1608 (57.5)	0.814	3 (0.1)
WBC > 10,000/mm^3^	1165 (21.3)	607 (21.7)	0.713	2 (0.1)
Platelet count < 150,000/mm^3^	857 (15.7)	390 (14.0)	**0.038**	3 (0.1)
Albumin level < 3.0 g/dL	665 (12.2)	310 (11.1)	0.158	133 (4.8)
INR ≥ 1.5	444 (8.1)	227 (8.1)	0.966	351 (12.6)
Hematocrit ≤ 35%	2939 (53.8)	1517 (54.3)	0.691	8 (0.3)
BUN ≥ 25 mg/dL	1409 (25.8)	665 (23.8)	0.053	25 (0.9)
Creatinine ≥ 1.5 mg/dL	669 (12.3)	328 (11.7)	0.544	28 (1.0)
CRP ≥ 1.0 mg/dL	4236 (77.6)	2198 (78.6)	0.287	83 (3.0)
Home medication				
Low-dose aspirin	1084 (19.9)	549 (19.6)	0.838	0
Antiplatelet drug (non-aspirin)	742 (13.6)	398 (14.2)	0.419	0
Warfarin	377 (6.9)	202 (7.2)	0.585	0
Direct oral anticoagulant	341 (6.2)	158 (5.7)	0.305	0
NSAIDs	637 (11.7)	308 (11.0)	0.401	0
Acetaminophen	130 (2.4)	64 (2.3)	0.818	0
Corticosteroid	290 (5.3)	163 (5.8)	0.332	0
Comorbidity				
Previous diverticular bleeding	804 (14.7)	446 (16.0)	0.145	1 (0.0)
Hypertension	2941 (53.9)	1522 (54.5)	0.624	0
Dyslipidemia	1424 (26.1)	708 (25.3)	0.473	0
Diabetes mellitus	1032 (18.9)	479 (17.1)	0.051	0
Diabetes complication	173 (3.2)	94 (3.4)	0.646	0
Hemiplegia	149 (2.7)	75 (2.7)	0.943	1 (0.0)
Cerebrovascular disease	775 (14.2)	387 (13.8)	0.688	0
Chronic obstructive pulmonary disease	161 (2.9)	72 (2.6)	0.362	0
Dementia	316 (5.8)	149 (5.3)	0.726	1 (0.0)
Collagen disease	235 (4.3)	110 (3.9)	0.450	0
Ischemic heart disease	813 (14.9)	422 (15.1)	0.819	0
Heart failure	427 (7.8)	232 (8.3)	0.466	1 (0.0)
Previous peptic ulcer	397 (7.3)	179 (6.4)	0.144	0
Renal failure	751 (13.8)	368 (13.2)	0.476	1 (0.0)
Peripheral arterial disease	210 (3.8)	111 (4.0)	0.810	0
Chronic hepatitis	128 (2.3)	58 (2.1)	0.481	0
Cirrhosis	113 (2.1)	57 (2.0)	1.000	0
Blood malignancy and nonmetastatic solid cancer^†^	758 (13.9)	384 (13.7)	0.866	0
Metastatic cancer	140 (2.6)	77 (2.8)	0.611	0
Diagnostic procedure				
CT	3799 (69.6)	2,018 (72.2)	**0.014**	0
Therapeutic procedures				
Endoscopic treatment	1751 (32.1)	896 (32.1)	1.000	0
Interventional radiology	106 (1.9)	53 (1.9)	0.933	0
Surgery	90 (1.6)	31 (1.1)	0.053	0
Blood transfusion during hospitalization	1618 (29.6)	829 (30.0)	1.000	0
The final diagnosis of hematochezia				
Colonic diverticular bleeding	3137 (57.5)	1672 (59.8)	0.143	0
Rectal ulcer	152 (2.8)	91 (3.3)	0.242	0
Angioectasia	82 (1.5)	34 (1.2)	0.324	0
Upper gastrointestinal bleeding	109 (2.0)	39 (1.4)	0.054	0
Small intestinal bleeding	132 (2.4)	64 (2.3)	0.760	0
Malignancy	133 (2.4)	54 (1.9)	0.317	0
Others	382 (7.0)	192 (6.9)	0.855	0
In-hospital outcomes				
Length of hospital stay ≥ 8 days	2514 (46.1)	1297 (46.4)	0.436	0
Rebleeding during hospitalization	774 (14.2)	378 (13.5)	0.421	0
Thromboembolism	40 (0.7)	15 (0.5)	0.321	0
Death within 30-day	51 (0.9)	23 (0.8)	0.712	0

*BMI* body mass index, *BUN* blood urea nitrogen, *CI* confidence interval, *CRP* C-reactive protein, *CT* computed tomography, *INR* international normalized ratio, *NSAIDs* nonsteroidal anti-inflammatory drugs, *WBC* white blood cell count.

*Bold values indicate *P* < 0.05.

^†^Blood malignancy was included with the comorbidity of leukemia and lymphoma.

Cox regression analysis was determined based on 8–10 events per predictor variable^24,25^. The logistic regression model was employed to calculate crude odds ratios (ORs), adjusted ORs, and 95% confidence intervals (CIs) for 30-day mortality. Model selection for multivariable analyses of 30-day mortality was conducted using the stepwise method (*P*-value to enter = 0.10 and *P*-value to stay = 0.05). The final model was identified based on the Akaike information criterion, and the goodness of fit was examined using the Hosmer–Lemeshow test^26^. Accordingly, the predictor weight was calculated based on model coefficients. Each point value in the scoring system was assigned to each rounded coefficient value. The area under the receiver operating characteristic curve (ROC-AUC) data were used to evaluate the score’s discriminatory power. The validation cohort was used to evaluate the validity of prediction scores. Based on the number of score points, patients were divided into three groups, and the mortality of each group was compared using Fisher’s exact test with Bonferroni correction. We compared the scores developed in this study with three previously reported ALGIB scores (Sengupta, Oakland, and NOBLADS) and incorporated the Charlson Comorbidity Index (CCI) as a common measure for mortality prediction to evaluate the prognostic utility of comorbidities^1,2,12,19^. We compared the ROC-AUC using the DeLong test^27^. The specifics of each score are shown in Supplementary Table 4.

We created a scoring system for predicting 1-year mortality following admission using baseline characteristics and in-hospital management factors. In the univariate analysis, the Cox proportional hazards model was used to assess the predictors of 1-year mortality. This analysis was used to produce crude hazard ratios (HRs) and 95% CIs. Model selection, the final model, the weight of the predictor, and the value of scoring system points were determined employing the same steps as the first analysis. The accuracy of the predictive model for 1-year mortality was assessed by the c-statistic using Harrell’s technique^28^. The mortality of each group was compared using the Kaplan–Meier method and the Cox proportional hazards model. Subjects were separated into three groups depending on the scores. Each risk factor for 1-year mortality was broken down into three distinct categories in the subgroup analysis to analyze the relationships with long-term death using the Cox proportional hazards model. We followed the TRIPOD statement for reporting this clinical prediction model study^29^. R version 4.2.2 was used to conduct all statistical tests.

## Results

### Predictive factors for 30-day mortality and predictive score

The analysis included 8254 patients (median [interquartile range] age, 74 [63–82] years; 4973 [60.2%] men). Except for platelet counts of < 150,000/mm^3^ and the use of computed tomography for diagnosis, the baseline characteristics of the derivation (n = 5459) and validation (n = 2795) cohorts were similar (Table 1).

**Figure 2:**
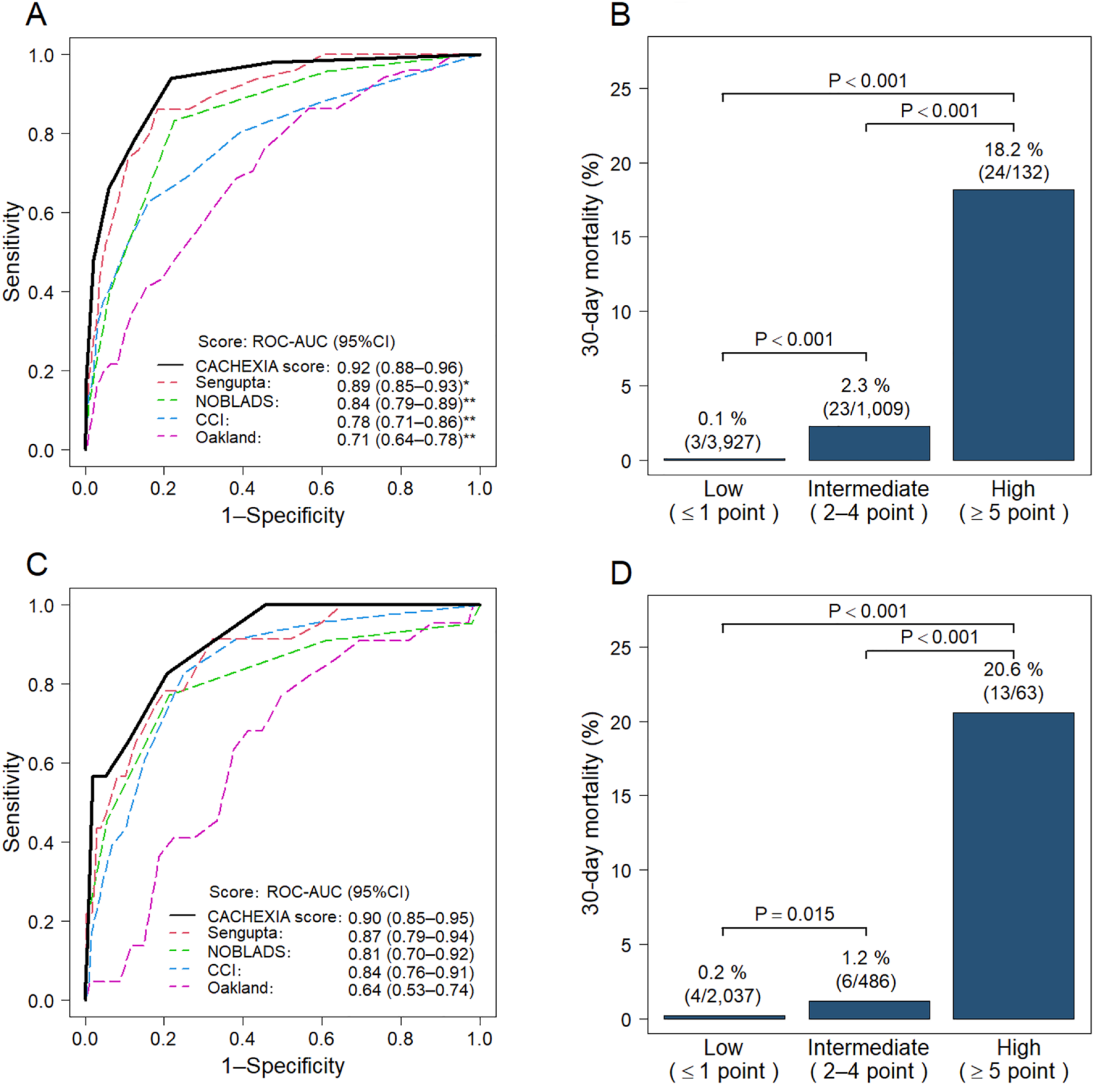
Predictive ability of the CACHEXIA score for 30-day mortality.** (A)** Comparison of scoring methods in the derivation cohort (n = 5459). **(B)** 30-day death rates by risk category for the derivation cohort (n = 5459). **(C)** Comparison of scoring systems in the validation cohort (n = 2795). **(D)** 30-day mortality rates by risk category in the validation cohort (n = 2795). *P* values were determined using Fisher’s exact test with Bonferroni correction** (B,D)**. *P value was 0.003, **P value was < 0.001. *ROC-AUC* receiver operator characteristic curves of the area under the curve, *CCI* Charlson comorbidity index.

In the derivation cohort, 51 (0.9%) of 5459 patients died within 30 days; 7 (0.1%) patients died of bleeding-related causes (Supplementary Table 5). The univariate analysis revealed 26 baseline parameters linked to 30-day mortality. Multivariate logistic regression analysis identified the following six factors as risk factors for 30-day mortality: performance status (PS) ≥ 2, albumin level < 3.0 g/dL, blood urea nitrogen (BUN) ≥ 25 mg/dL, C-reactive protein (CRP) ≥ 1.0 mg/dL, comorbid metastatic cancer, and cirrhosis (Table 2). Based on the coefficients from the multivariate analysis, we created a novel weighted score for predicting 30-day mortality (maximum 11 points) utilizing these six factors (Table 2). For the derivation cohort, the ROC-AUC of the new score was 0.92 (95% CI 0.88–0.96), which was significantly higher than existing clinical risk scores (ROC-AUC: Sengupta, 0.89; NOBLADS, 0.84; CCI, 0.78; Oakland, 0.71) (Fig. 2A). Based on the novel score, patients in the derivation cohort were categorized into low-score ≤ 1 (n = 3927), medium-score 2–4 (n = 1009), and high-score ≥ 5 (n = 132) groups. The 30-day mortality rates for the low-, medium-, and high-score groups were 0.1% (n = 5), 2.3% (n = 23), and 18.2% (n = 24), respectively (*P* < 0.001 for each comparison) (Fig. 2B).

**Table 2:** Predictors of 30-day mortality by logistic regression model (derivation cohort, n = 5459).

Characteristics	Crude odds ratio (95% CI)	*P* value*	Adjusted odds ratio (95% CI)	Coefficient (95% CI)	*P* value*	Score points
Age ≥ 70 years	2.25 (1.18–4.32)	**0.014**				
Sex (male)	1.20 (0.68–2.14)	0.533				
BMI ≤ 18.5	2.16 (1.09–4.27)	**0.027**				
Current drinker	0.42 (0.21–0.84)	**0.014**				
Current smoker	1.35 (0.66–2.75)	0.412				
Performance status ≥ 2	9.97 (5.67–17.51)	** < 0.001**	5.54 (2.54–12.05)	1.71 (0.93–2.49)	** < 0.001**	2
Hemodynamics						
Systolic blood pressure ≤ 100 mmHg	4.86 (2.78–8.50)	** < 0.001**				
Heart rate ≥ 100/min	1.08 (0.55–2.11)	0.825				
Symptom						
Altered mental status	2.71 (1.26–5.82)	**0.011**				
Abdominal pain	1.00 (0.48–2.06)	0.992				
Diarrhea	0.67 (0.32–1.44)	0.306				
Laboratory data						
Hemoglobin < 12 g/dL	3.43 (1.67–7.06)	** < 0.001**				
WBC > 10,000/mm^3^	2.83 (1.62–4.94)	** < 0.001**				
Platelet count < 150,000/mm^3^	2.72 (1.51–4.88)	** < 0.001**				
Albumin level < 3.0 g/dL	20.52 (10.85–38.82)	** < 0.001**	5.45 (2.23–13.31)	1.70 (0.80–2.59)	** < 0.001**	2
INR ≥ 1.5	2.81 (1.43–5.52)	**0.003**				
Hematocrit ≤ 35%	3.13 (1.60–6.12)	** < 0.001**				
BUN ≥ 25 mg/dL	5.38 (2.99–9.49)	** < 0.001**	2.57 (1.19–5.57)	0.95 (0.17–1.72)	**0.016**	1
Creatinine ≥ 1.5 mg/dL	3.62 (2.01–6.52)	** < 0.001**				
CRP ≥ 1.0 mg/dL	10.42 (5.53–19.62)	** < 0.001**	3.35 (1.41–7.92)	1.21 (0.35–2.07)	**0.006**	1
Home medication						
Low-dose aspirin	1.39 (0.74–2.61)	0.313				
Antiplatelet drug (non-aspirin)	0.85 (0.36–1.99)	0.702				
Warfarin	2.17 (0.97–4.84)	0.060				
Direct oral anticoagulant	0.61 (0.15–2.52)	0.495				
NSAIDs	2.13 (1.03–4.40)	**0.041**				
Acetaminophen	1.68 (0.41–7.00)	0.474				
Corticosteroid	1.14 (0.35–3.60)	0.855				
Comorbidity						
Previous diverticular bleeding	0.23 (0.06–0.97)	**0.044**				
Hypertension	0.82 (0.47–1.43)	0.485				
Dyslipidemia	0.69 (0.34–1.38)	0.292				
Diabetes mellitus	1.32 (0.69–2.54)	0.398				
Diabetes complication	1.93 (0.59–6.25)	0.275				
Hemiplegia	3.97 (1.55–10.14)	**0.004**				
Cerebrovascular disease	1.87 (0.98–3.59)	0.059				
Chronic obstructive pulmonary disease	3.66 (1.44–9.34)	**0.007**				
Dementia	3.08 (1.44–6.60)	**0.004**				
Collagen disease	1.39 (0.43–4.51)	0.579				
Ischemic heart disease	0.91 (0.41–2.02)	0.814				
Heart failure	2.56 (1.24–5.29)	**0.011**				
Previous peptic ulcer	1.39 (0.55–3.52)	0.486				
Renal failure	2.17 (1.15–4.08)	**0.017**				
Peripheral arterial disease	2.76 (1.09–7.02)	**0.033**				
Chronic hepatitis	1.71 (0.41–7.11)	0.460				
Cirrhosis	5.33 (2.08–13.69)	** < 0.001**	4.72 (1.44–15.49)	1.55 (0.36–2.74)	**0.011**	2
Blood malignancy and nonmetastatic solid cancer^†^	6.13 (3.52–10.68)	** < 0.001**				
Metastatic cancer	14.23 (7.40–27.36)	** < 0.001**	10.84 (4.56–25.94)	2.38 (1.51–3.26)	** < 0.001**	3

*BMI* body mass index, *BUN* blood urea nitrogen, *CI* confidence interval, *CRP* C-reactive protein, *INR* international normalized ratio, *NSAIDs* nonsteroidal anti-inflammatory drugs, *WBC* white blood cell count.

*Bold values indicate *P* < 0.05.

^†^Blood malignancy was included with the comorbidity of leukemia and lymphoma.

In the validation cohort, the ROC-AUC for the new score was 0.90 (95% CI 0.85–0.95) (Fig. 2C). Patients in low-, medium-, and high-score groups had 30-day mortality rates of 0.2% (n = 4), 1.2% (n = 6), and 20.6% (n = 13), respectively. The mortality rates were significantly higher in the medium-score group compared with the low-score group and in the high-score group compared with the low- and medium-score groups (low vs. medium score, *P* = 0.003; high vs. medium/low scores, *P* < 0.001) (Fig. 2D). The calibration plot of actual probability compared with the predicted probability of 30-day mortality showed an intercept of − 0.03, a slope of 0.90, and a mean absolute prediction error of 0.005, suggesting acceptable calibration (Supplementary Fig. 1A).

### Predictive factors for 1-year mortality and predictive score

Mortality within 1 year was assessed in 6084 cases after excluding the 2170 cases (Fig. 1). The characteristics and in-hospital care of the derivation and validation cohorts were similar, except for five factors: platelet count < 150,000/mm^3^, diabetes mellitus, the use of CT for diagnosis, surgery for hemostasis, and bleeding due to cancer (Supplementary Table 3). In the derivation cohort, 163 (3.0%) of 4030 patients died within 1 year. Univariate analysis showed that 34 factors were associated with 1-year mortality among the baseline characteristics and in-hospital management data. The Cox proportional hazards model showed 10 risk factors for 1-year mortality, including PS ≥ 2, albumin level < 3.0 g/dL, BUN ≥ 25 mg/dL, CRP ≥ 1.0 mg/dL, comorbid metastatic cancer, cirrhosis, body mass index (BMI) < 18.5, blood transfusion during hospitalization, blood malignancy and solid cancer, and bleeding from malignancy (hematochezia due to cancer) (Table 3). Based on the coefficients from the multivariate analysis, we created a novel weighted score for predicting 1-year mortality (maximum 17 points) (Table 3).

**Table 3:** Predictors of 1-year mortality by Cox proportional hazards model (derivation cohort, n = 4030).

Characteristics	Crude hazard ratio (95% CI)	*P* value*	Adjusted hazard ratio (95% CI)	Coefficient (95% CI)	*P* value*	Score points
Age ≥ 70 years	1.98 (1.39–2.84)	** < 0.001**				
Sex (male)	1.17 (0.85–1.61)	0.347				
BMI ≤ 18.5	4.01 (2.89–5.55)	** < 0.001**	1.80 (1.21–2.68)	0.59 (1.89–0.99)	**0.004**	1
Current drinker	0.75 (0.53–1.06)	0.102				
Current smoker	0.69 (0.43–1.12)	0.136				
Performance status ≥ 2	4.29 (3.09–5.96)	** < 0.001**	2.22 (1.49–3.31)	0.80 (0.40–1.20)	** < 0.001**	2
Hemodynamics						
Systolic blood pressure ≤ 100 mmHg	2.92 (2.08–4.09)	** < 0.001**				
Heart rate ≥ 100/min	1.61 (1.14–2.27)	**0.007**				
Symptom						
Altered mental status	1.18 (0.64–2.18)	0.588				
Abdominal pain	0.81 (0.53–1.23)	0.319				
Diarrhea	1.17 (0.70–1.95)	0.562				
Laboratory data						
Hemoglobin < 12 g/dL	3.91 (2.55–6.00)	** < 0.001**				
WBC > 10,000/mm^3^	1.26 (0.88–1.80)	0.210				
Platelet count < 150,000/mm^3^	2.66 (1.92–3.69)	** < 0.001**				
Albumin level > 3.0 g/dL	6.77 (4.95–9.26)	** < 0.001**	2.03 (1.33–3.09)	0.71 (0.28–1.13)	**0.001**	1
INR ≥ 1.5	1.99 (1.33–2.98)	** < 0.001**				
Hematocrit ≤ 35%	3.61 (2.44–5.33)	** < 0.001**				
BUN ≥ 25 mg/dL	3.24 (2.38–4.40)	** < 0.001**	1.90 (1.32–2.73)	0.64 (0.28–1.01)	**0.001**	1
Creatinine ≥ 1.5 mg/dL	3.35 (2.42–4.64)	** < 0.001**				
CRP ≥ 1.0 mg/dL	3.08 (2.27–4.19)	** < 0.001**	1.70 (1.15–2.51)	0.53 (0.14–0.92)	**0.008**	1
Home medication						
Low-dose aspirin	1.03 (0.71–1.49)	0.884				
Antiplatelet drug (non-aspirin)	0.96 (0.62–1.49)	0.842				
Warfarin	1.16 (0.68–1.97)	0.584				
Direct oral anticoagulant	1.29 (0.73–2.27)	0.381				
NSAIDs	2.03 (1.35–3.04)	** < 0.001**				
Acetaminophen	2.32 (1.19–4.55)	**0.014**				
Corticosteroid	2.02 (1.25–3.25)	**0.004**				
Comorbidity						
Previous diverticular bleeding	0.34 (0.18–0.67)	** < 0.001**				
Hypertension	0.72 (0.53–0.98)	**0.038**				
Dyslipidemia	0.77 (0.53–1.10)	0.148				
Diabetes mellitus	1.26 (0.89–1.81)	0.197				
Diabetes complication	1.57 (0.80–3.08)	0.187				
Hemiplegia	1.47 (0.60–3.57)	0.399				
Cerebrovascular disease	1.16 (0.73–1.70)	0.611				
Chronic obstructive pulmonary disease	1.73 (0.88–3.39)	0.110				
Dementia	2.72 (1.65–4.49)	** < 0.001**				
Collagen disease	1.61 (0.91–2.83)	0.102				
Ischemic heart disease	0.81 (0.52–1.27)	0.354				
Heart failure	2.40 (1.62–3.57)	** < 0.001**				
Previous peptic ulcer	1.48 (0.91–2.41)	0.119				
Renal failure	2.16 (1.53–3.04)	** < 0.001**				
Peripheral arterial disease	1.49 (0.79–2.83)	0.219				
Chronic hepatitis	1.74 (0.85–3.54)	0.128				
Cirrhosis	5.64 (3.41–9.32)	** < 0.001**	4.44 (2.46–8.01)	1.49 (0.90–2.08)	** < 0.001**	3
Blood malignancy and nonmetastatic solid cancer^†^	7.82 (5.72–10.68)	** < 0.001**	3.03 (1.97–4.66)	1.11 (0.68–1.54)	** < 0.001**	2
Metastatic cancer	14.96 (10.58–21.16)	** < 0.001**	4.48 (2.75–7.31)	1.50 (1.01–1.99)	** < 0.001**	3
Diagnostic procedure						
CT	0.80 (0.58–1.09)	0.161				
Therapeutic procedures						
Endoscopic treatment	0.77 (0.53–1.12)	0.171				
Interventional radiology	1.03 (0.33–3.21)	0.965				
Surgery	2.34 (1.10–4.99)	**0.028**				
Blood transfusion during hospitalization	3.51 (2.58–4.77)	** < 0.001**	1.92 (1.37–2.82)	0.65 (0.27–1.04)	**0.001**	1
The final diagnosis of hematochezia						
Colonic diverticular bleeding	0.34 (0.25–0.48)	** < 0.001**				
Rectal ulcer	5.43 (3.19–9.24)	** < 0.001**				
Angioectasia	2.23 (0.99–5.05)	0.054				
Upper gastrointestinal bleeding	3.59 (1.89–6.80)	** < 0.001**				
Small intestinal bleeding	2.24 (1.10–4.56)	**0.026**				
Malignancy	6.43 (4.17–9.92)	** < 0.001**	2.61 (1.56–4.36)	0.96 (0.45–1.47)	** < 0.001**	2
Others	2.61 (1.71–4.00)	** < 0.001**				
In-hospital outcomes						
Length of hospital stay ≥ 8 days	2.43 (1.74–3.38)	** < 0.001**				
Rebleeding during hospitalization	1.01 (0.65–1.57)	0.956				
Thromboembolism	4.54 (2.01–10.27)	** < 0.001**				

*BMI* body mass index, *BUN* blood urea nitrogen, *CI* confidence interval, *CRP* C-reactive protein, *CT* computed tomography, *INR* international normalized ratio, *NSAIDs* nonsteroidal anti-inflammatory drugs, *WBC* white blood cell count.

*Bold values indicate *P* < 0.05.

^†^Blood malignancy was included with the comorbidity of leukemia and lymphoma.

**Figure 3:**
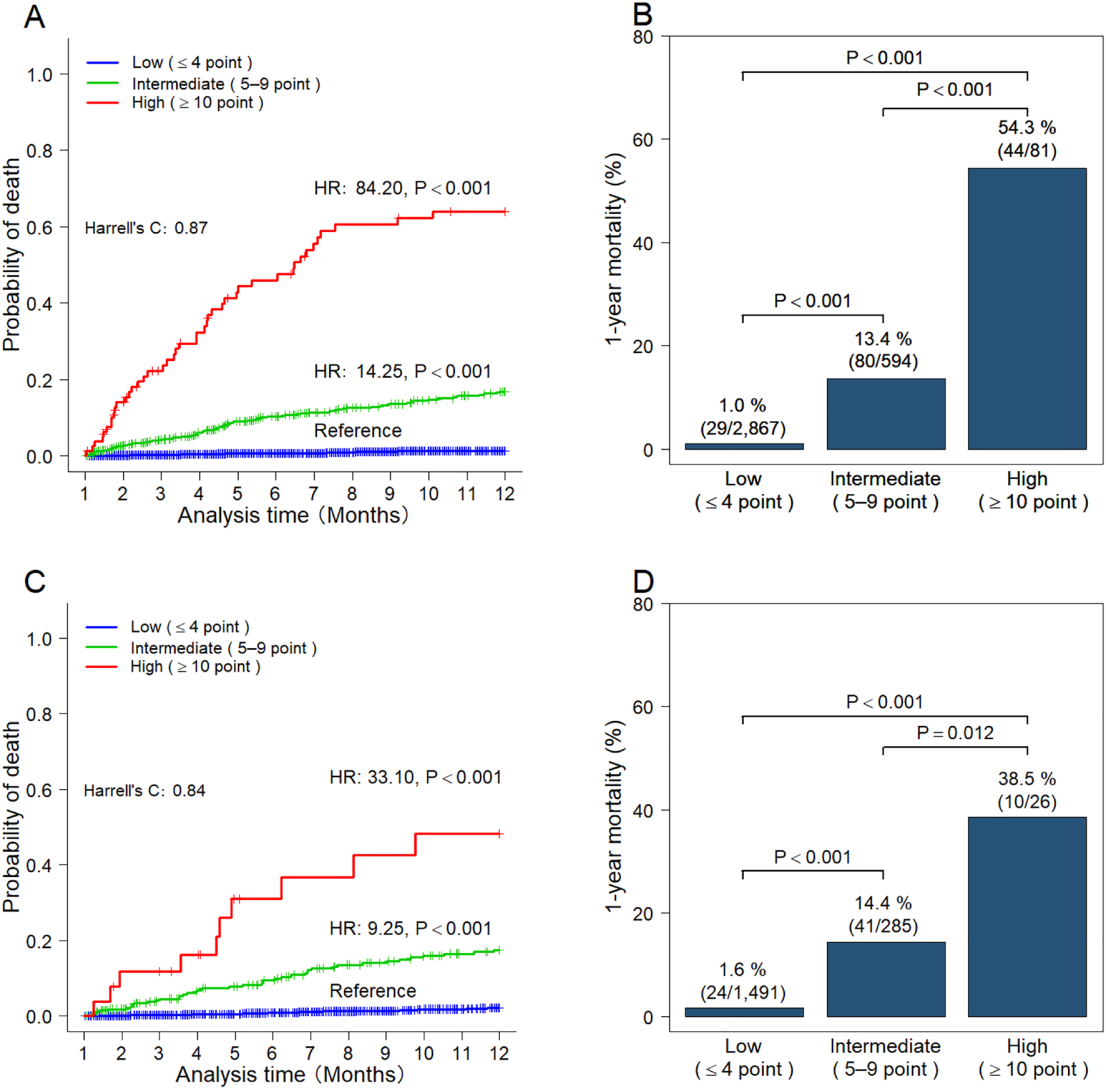
Predictive ability of the CACHEXIA score for 1-year mortality. **(A)** Cumulative probability of mortality according to risk category in the derivation cohort (n = 4030). **(B)** One-year mortality rates by risk category in the derivation cohort (n = 4030). **(C)** The cumulative death probability according to risk category in the validation cohort (n = 2054). **(D)** One-year mortality rates by risk category in the validation cohort (n = 2054). *P* values were calculated using the Cox proportional hazards model **(A,C)** and Fisher’s exact test with Bonferroni correction **(B,D)**. Cases with missing data were excluded from the full case analysis **(B,D)**. *ROC-AUC* receiver operator characteristic curves of the area under the curve, *CCI* Charlson comorbidity index.

In the derivation cohort, the c-statistic for the novel score was 0.87 (95% CI 0.84–0.90). Patients were categorized into low-score ≤ 4 (n = 2867), medium-score 5–9 (n = 594), and high-score ≥ 10 (n = 81) groups. A log-rank test revealed that patients in the high-score group had significantly higher probabilities of death compared with patients in the low-score group (HR 84.20; 95% CI 52.6–137.9; *P* < 0.001) and the medium-score group (HR 14.25; 95% CI 9.32–21.8; *P* < 0.001) (Fig. 3A). The 1-year mortality rates for the low-, medium-, and high-score groups were 1.0%, 13.4%, and 54.3%, respectively (all: *P* < 0.001) (Fig. 3B).

The c-statistic for novel scores in the validation cohort was 0.84 (95% CI 0.80–0.89). Compared to the low-score group, the high-score group (HR 33.10; 95% CI 15.8–69.4; *P* < 0.001) and the intermediate-score group (HR 9.25; 95% CI 5.6–15.3; *P* < 0.001) had significantly higher probabilities of death (Fig. 3C). The 1-year mortality rates for the low-, medium-, and high-score groups were 1.6%, 14.4%, and 38.5%, respectively (low vs. intermediate and low vs. high: *P* < 0.001; intermediate vs. high: *P* = 0.012) (Fig. 3D). The calibration plot of actual probability compared with the predicted probability of 1-year survival showed a mean absolute prediction error of 0.006 (Supplementary Fig. 1B).

**Figure 4:**
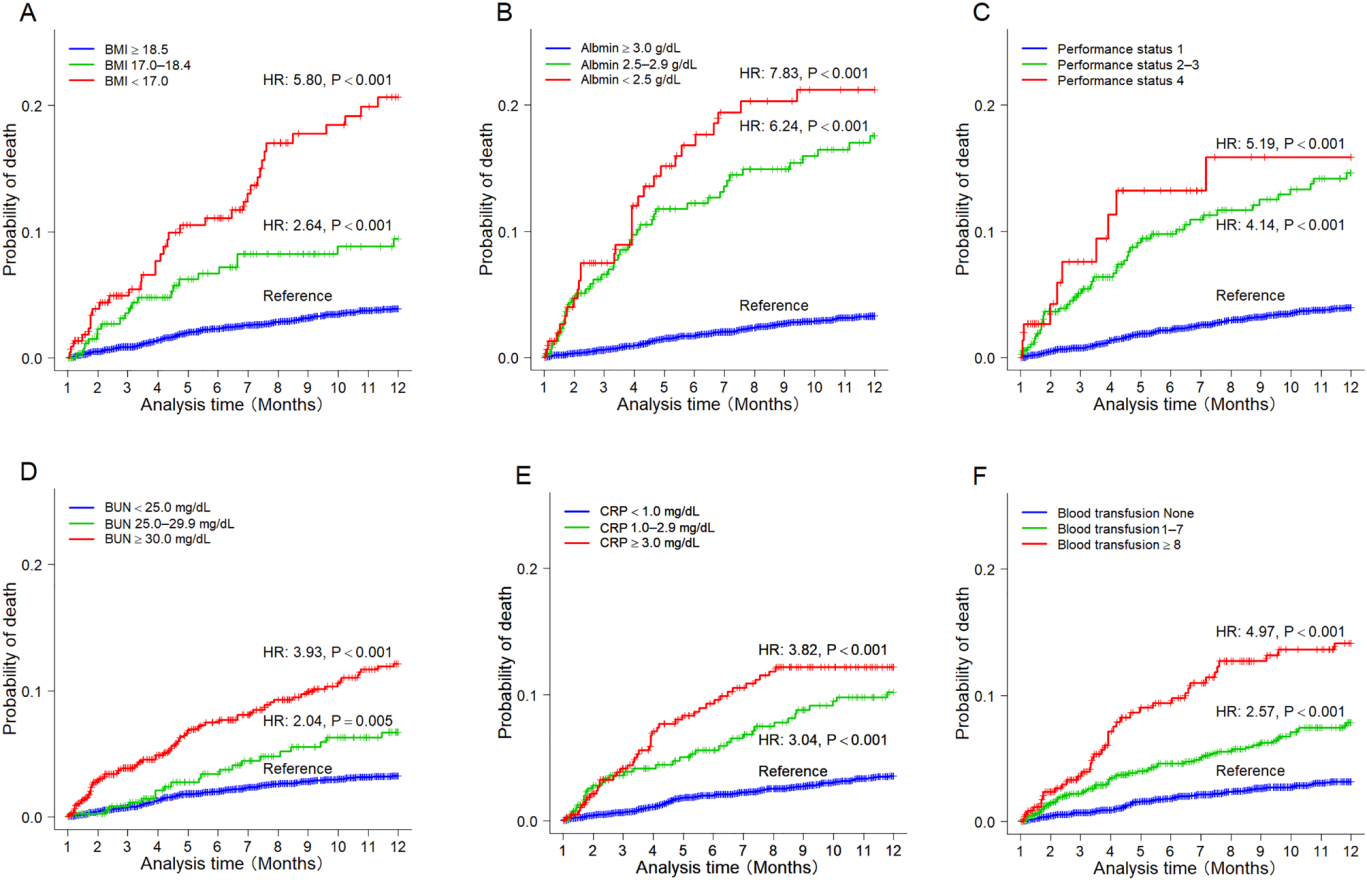
Cumulative probability of mortality according to risk factors using the Kaplan–Meier method. **(A)** BMI (< 17.0, 17.0–18.4, and ≥ 18.5). **(B)** Albumin level (< 2.5, 2.5–2.9, and ≥ 3.0 g/dL). **(C)** Performance status (1, 2–3, and 4). **(D)** BUN (< 25.0, 25.0–29.9, and > 30.0 mg/dL). **(E)** CRP (< 1.0, 1.0–2.9, and ≥ 3.0 mg/dL). **(F)** Amount of blood transfusion (none, 1–7 units, and ≥ 8 units). *P* values were calculated using the Cox proportional hazards model. *BMI* body mass index, *BUN* blood urea nitrogen, *CRP* C-reactive protein.

Group analysis was carried out to investigate the association between 1-year mortality and various factors, which were split into three categories. In the Cox proportional hazards model, a decrease in BMI (< 17.0, 17.0–18.4, and ≥ 18.5) and albumin levels (< 2.5, 2.5–2.9, and ≥ 3.0 g/dL) significantly increased the probability of 1-year mortality (all: *P* < 0.001) (Fig. 4A,B). In contrast, increases in PS (1, 2, 3, and 4), BUN (< 25.0, 25.0–29.9, > 30.0 mg/dL), CRP (< 1.0, 1.0–2.9, and > 3.0 mg/dL), and the amount of blood transfusion (none, 1–7 units, and 8 units) significantly increased the probability of 1-year mortality (all: *P* < 0.001) (Fig. 4C–F).

## Discussion

To calculate long-term mortality risks, we analyzed information from a multicenter trial that included 8254 patients with acute hematochezia who required emergency hospitalization. The 30-day and 1-year mortality rates were low, at 0.9% and 3.0%, respectively. We created a highly accurate (c-index 0.87) long-term prognostic scoring system called the CACHEXIA score (Cancer including metastasis tumor, blood tumor, and bleeding from tumor, Albumin, Cirrhosis, High PS, EXtremely thin (i.e., low BMI), Increased CRP and BUN, Anemia (i.e., blood transfusion)). Each predictor was assigned a score based on adjusted regression coefficients, and risk was stratified by summing these scores. The 1-year mortality rates for patients with low-, medium-, and high-risk CACHEXIA scores were 1.0%, 13.4%, and 54.3%, respectively. A few of these variables also helped forecast the short-term prognosis. Our results demonstrate that cachexia-related factors rather than bleeding-related factors are highly correlated with prognosis in patients with acute hematochezia. The innovative predictive score facilitates accurate stratification of the high-risk group despite the low mortality rate of patients with acute hematochezia.

Aoki et al.^9^ (n = 342) and Arroja et al.^10^ (n = 364) reported mortality rates of 4.2% and 2.2% 1 year post-hospitalization, respectively, for the long-term prognosis of ALGIB, which are comparable to that of the present study (3.0%). Thus, results of previous studies were validated by our large cohort. Regarding short-term outcomes (death within 30 days or in-hospital mortality), previously reported risk factors include age, comorbid illnesses, hypoalbuminemia, and low BMI, which, apart from age, do not contradict the results of this study^5,7,11,30^. Strate et al., based on an analysis of over 200,000 cases using ICD codes, pointed out that age, comorbidities, and bleeding while hospitalized for other diseases are strongly correlated with in-hospital mortality. In contrast, they noted that diverticular bleeding, a common cause of severe bleeding, is not associated with systemic illness and therefore does not contribute to mortality risk^11^. The short-term mortality rate (0.9%) in this study was lower than the mortality rate in a study conducted by Sengupta et al. (10.9%). However, predictors of mortality help explain this disparity. Metastatic tumors and cirrhosis accounted for 12.3% and 6.6% of patients in the previous analysis, respectively, which were higher than the rates in the present study (2.6% and 2.1%, respectively). Thus, the prevalence of prognostic indicators may have a significant impact on the mortality rate.

Risk factors identified in our study included many diseases with poor progressive prognosis, such as malignant disease and cirrhosis. Notably, the findings showed that cachexia, a poor prognostic condition, had a significant impact on the long-term prognosis of patients with hematochezia. Cachexia is a debilitating condition characterized by poor nutritional status, weight loss, and decreased BMI as objective indicators^31,32^. Furthermore, increased catabolism, cancer treatment resistance, and elevated PS are associated with refractory cancer cachexia^33^. Cancer cachexia is associated with systemic inflammation, and CRP levels correlate with shorter survival in patients with advanced cancer^22^. Therefore, cachexia is closely associated with unfavorable prognostic variables found in the current study, including high PS, low albumin level, high CRP, and low BMI.

The CACHEXIA score developed in our study is a long-term prognostic score encompassing short-term prognostic factors. This score is used to predict long-term prognosis in patients with acute hematochezia. Furthermore, this new score has been shown to be a more accurate predictive tool for short-term mortality than other ALGIB prognostic scores. Previously, Nagata et al. reported that patients with gastrointestinal bleeding have a poorer long-term prognosis than patients with non-gastrointestinal bleeding^34^. Stratified data on long-term prognosis are scarce, and prognostication after gastrointestinal bleeding is essential in managing patients with multiple diseases. Patients highly at risk of death may refuse invasive cancer treatments and receive more comprehensive medical care. Patients with high scores are expected to have a poor prognosis and may be candidates for mortality prevention strategies. Pharmacological therapies, nutrition therapy, exercise, and psychosocial interventions are vital in preventing the advancement of cachexia^35–38^. Conversely, even with repeated bleeding, patients with low scores would be expected to have a good prognosis and may be candidates for interventions against bleeding.

The strength of this study was its ability to assess long-term results despite the copious amounts of data gathered during the medical record survey. However, this study had several limitations. First, this research was retrospective and subject to selection bias. For instance, our investigation was limited to cases of hematochezia with emergency admissions. Assuming that patients with life-threatening lower gastrointestinal bleeding are hospitalized emergently, we focused on patients requiring emergency admission. However, admission criteria vary by country and facility, reducing external validity. The Oakland score is widely used globally as a severity score for lower gastrointestinal bleeding and is specified in UK guidelines as a criterion for hospitalization, suggesting its widespread use in many countries and facilities. In our study, we could not confirm that the Oakland score was used as a criterion for hospitalization. Instead, we used the ROC-AUC to compare the CACHEXIA score with the Oakland score, examining which is more suitable for predicting mortality. Additionally, our study did not include a comparison with cases manageable on an outpatient basis, leaving doubts about the appropriateness of using the CACHEXIA score for all cases of hematochezia. In fact, all chronic diseases included in the CACHEXIA score variables are associated with a high 1-year mortality rate. Therefore, the CACHEXIA score might not be a prognostic tool specialized for gastrointestinal bleeding. Second, all participating institutions were Japanese institutions with a sizable number of beds and access to endoscopy. As a result, the generalizability of our scores in different situations, including nonemergency hospitals and hospitals in other countries, should be confirmed. Thirdly, in this study, 2096 cases were lost to follow-up from the 1-year cohort. With ALGIB cases, there tends to be a cessation of follow-up visits once the hemorrhage stops and the anemia improves, as patients become asymptomatic. This is considered one of the reasons why long-term prognosis studies of lower gastrointestinal bleeding are difficult. To resolve such issues, retrospective studies have their limitations. Therefore, it is desirable to conduct prospective studies to carry out more rigorous and high-quality investigations.

In conclusion, our nationwide long-term data indicate the prognosis of patients with acute hematochezia is related more closely to chronic diseases and associated cachexia than to bleeding severity, diagnosis, and treatment. Although the overall mortality rate was low, based on our novel predictive score, high-risk patients should be followed up at the medical facility even after discharge from ALGIB hospitalization.

### Supplementary Information

Supplementary Information.

## Data Availability

Access to the data supporting the results of this study will be requested and reviewed with the principal investigator of this study through the corresponding author. The data are not available to the public due to privacy and ethical restrictions.
