# Supplementary material for: A novel prediction tool for mortality in patients with acute lower gastrointestinal bleeding requiring emergency hospitalization: a large multicenter study
Source: Sci Rep. 2024 Mar 4;14:5367. doi: 10.1038/s41598-024-55889-7 (PMC10912311; doi:10.1038/s41598-024-55889-7)
Supplement: Supplementary file 1 — Supplementary Information. [file 41598_2024_55889_MOESM1_ESM.pdf]

Supplementary Table 1. Affiliations, ethics committee approval number, and number of cases at the 49 participating hospitals in the CODE BLUE J-study

No.	Prefecture	Institutions	Department	Ethics committee approval number
1	Tokyo	Tokyo Medical University	Department of Gastroenterological Endoscopy	T20190244
2	Tokyo	National Center for Global Health and Medicine	Department of Gastroenterology and Hepatology	3539
3	Tokyo	Tokyo Shinagawa Hospital	Department of Gastroenterology	20-A-04
4	Tokyo	Nippon Medical School, Graduate School of Medicine	Department of Gastroenterology	B-2020-147
5	Chiba	Chiba Hokusoh Hospital, Nippon Medical School	Department of Gastroenterology	802
6	Saga	Saga-Ken Medical Centre Koseikan	Department of Gastroenterology	20-01-01-03
7	Tokyo	St. Luke's International University	Department of Gastroenterology	20-R012
8	Okayama	Kawasaki Medical School	Division of Endoscopy and Ultrasonography, Department of Clinical Pathology and Laboratory Medicine	3890
9	Okayama	Kawasaki Medical School General Medical Center	Division of Endoscopy and Ultrasonography, Department of Clinical Pathology and Laboratory Medicine	3890
10	Ibaraki	University of Tsukuba	Department of Gastroenterology and Division of Endoscopic Center	R02-030
11	Tokyo	Tokyo Metropolitan Bokutoh Hospital	Department of Gastroenterology	02-024
12	Kanagawa	Saiseikai Yokohamashi Tobu Hospital	Emergency and Critical Care Center	20200030
13	Tokyo	The University of Tokyo	Department of Gastroenterology	2020067NI
14	Tokyo	Toranomon Hospital	Department of Gastroenterology	2021

15	Aichi	Nagoya University Hospital	Department of Endoscopy	2020-0152
16	Hiroshima	Hiroshima City Asa Citizens Hospital	Department of Gastroenterology	02-1-24
17	Fukuoka	National Hospital Organization Fukuokahigashi Medical Center	Department of Gastroenterology and Hepatology	2020-臨-2
18	Nara	Nara City Hospital	Department of Gastroenterology and Hepatology and Center for Digestive and Liver Diseases	NCH 倫 20-8
19	Niigata	Graduate School of Medical and Dental Sciences, Niigata University	Division of Gastroenterology	2020-0052
20	Kanagawa	St. Marianna University School of Medicine	Division of Gastroenterology and Hepatology, Department of Internal Medicine	4802
21	Oita	Oita University	Department of Gastroenterology	1845
22	Tokyo	Tokyo Saiseikai Central Hospital	Department of Internal Medicine	2020-015-01
23	Fukuoka	Fukuoka University Hospital	Department of Gastroenterological Endoscopy	U20-05-016
24	Fukuoka	Fukuoka University Chikushi Hospital	Department of Gastroenterology	C20-052
25	Osaka	Kitano Hospital, Tazuke Kofukai Medical Research Institute	Department of Gastroenterology and Hepatology	P200500400
26	Fukuoka	Graduate School of Medical Sciences, Kyushu University	Department of Medicine and Clinical Science	2020-289
27	Miyazaki	University of Miyazaki Hospital	Department of Gastroenterology and Hepatology, Center for Digestive Disease, and Division of Endoscopy	0-0734
28	Okinawa	University of the Ryukyus Hospital	Department of Endoscopy	1656
29	Okinawa	Naha City Hospital	Department of Gastroenterology	2004a4
30	Kagoshima	Kagoshima University Graduate School of Medical and Dental Sciences	Digestive and Lifestyle Diseases	200041 疫
31	Kagoshima	Kagoshima City Hospital	Department of Gastroenterology	2020-25
32	Kagoshima	Kagoshima Kouseiren Hospital	Department of Gastroenterology	215

33	Kagoshima	Kagoshima Medical Center	Department of Gastroenterology	2020-22
34	Kagoshima	Izumi General Medical Center	Department of Gastroenterology	60
35	Kagoshima	Kirishima City Medical Association Medical Center	Department of Gastroenterology	202005
36	Kagoshima	Kagoshima Prefectural Oshima Hospital	Department of Gastroenterology	97
37	Kyoto	National Hospital Organization Kyoto Medical Center	Department of Gastroenterology	20-020
38	Fukushima	Fukushima Medical University	Department of Gastroenterology	一般 2020-112
39	Tokyo	Tokyo Metropolitan Cancer and Infectious Diseases Center Komagome Hospital	Department of Gastroenterology	2503
40	Kanagawa	Kitasato University, School of Medicine	Department of Gastroenterology	C20-174
41	Osaka	Suita Municipal Hospital	Department of Gastroenterology and Hepatology	2020-研 2
42	Akita	Akita University Graduate School of Medicine	Department of Gastroenterology and Neurology	2491
43	Shizuoka	Japanese Red Cross Shizuoka Hospital	Department of Gastroenterology	2020-06
44	Aomori	Hirosaki University Hospital	Division of Endoscopy	2020-32
45	Kumamoto	Graduate School of Medical Sciences, Kumamoto University	Department of Gastroenterology and Hepatology	2040
46	Fukuoka	National Hospital Organization Kyushu Medical Center	Department of Gastroenterology	20C065
47	Iwate	Iwate Medical University	Department of Internal Medicine	MH2020-050
48	Yamaguchi	Shuto General Hospital	Department of Gastroenterology	H31-24
49	Saitama	National Defense Medical College	Department of Internal Medicine	4217

Supplementary Table 2. The list of predictors for 30-day and 1-year mortality scores in patients with acute hematochezia

Variables	30-day mortality score	1-year mortality score	Evidence of cut-off value
Age ≥ 70 y	○	○	Reference No. 5
Sex (male)	○	○	
BMI ≤ 18.5	○	○	Reference No. 5
Current drinker	○	○	
Current smoker	○	○	
Performance status ≥ 2	○	○	Reference No. 20
Hemodynamics			
Systolic blood pressure ≤ 100 mmHg	○	○	Reference No. 2
Heart rate ≥ 100/min	○	○	Reference No. 2
Symptom			

Altered mental status	○	○	
Abdominal pain	○	○	
Diarrhea	○	○	
Laboratory data			
Hemoglobin < 12 g/dL	○	○	Reference No. 21
WBC > 10000/mm^3	○	○	Reference No. 21
Platelet count < 150000/mm^3	○	○	Reference No. 2
Albumin level > 3.0 g/dL	○	○	Reference No. 2
INR ≥ 1.5	○	○	Reference No. 2
Hematocrit ≤ 35%	○	○	Calculated from hemoglobin values
BUN ≥ 25 mg/dL	○	○	Reference No. 21
Creatinine ≥ 1.5 mg/dL	○	○	Reference No. 2
CRP ≥ 1.0 mg/dL	○	○	Reference No. 22

Home medication		
Low-dose aspirin	○	○
Antiplatelet drug (non-aspirin)	○	○
Warfarin	○	○
Direct oral anticoagulant	○	○
NSAIDs	○	○
Acetaminophen	○	○
Corticosteroid	○	○
Comorbidity		
Previous diverticular bleeding	○	○
Hypertension	○	○
Dyslipidemia	○	○
Diabetes mellitus	○	○

Diabetes complication	◯	◯
Hemiplegia	◯	◯
Cerebrovascular disease	◯	◯
Chronic obstructive pulmonary disease	◯	◯
Dementia	◯	◯
Collagen disease	◯	◯
Ischemic heart disease	◯	◯
Heart failure	◯	◯
Previous peptic ulcer	◯	◯
Renal failure	◯	◯
Peripheral arterial disease	◯	◯
Chronic hepatitis	◯	◯
Cirrhosis	◯	◯

Blood malignancy and nonmetastatic solid cancer[a]	○	○
Metastatic cancer	○	○
Diagnostic procedure		
CT	–	○
Therapeutic procedures		
Endoscopic treatment	–	○
Interventional radiology	–	○
Surgery	–	○
Blood transfusion during hospitalization	–	○
The final diagnosis of hematochezia		
Colonic diverticular bleeding	–	○
Rectal ulcer	–	○

Angioectasia	−	◯	
Upper gastrointestinal bleeding	−	◯	
Small intestinal bleeding	−	◯	
Malignancy	−	◯	
Others	−	◯	
In-hospital outcomes			
Length of hospital stay ≥ 8 days	−	◯	Reference No. 21
Rebleeding during hospitalization	−	◯	
Thromboembolism	−	◯	

Abbreviations: BMI, body mass index; BUN, blood urea nitrogen; CI, confidence interval; CRP, C-reactive protein; CT, computed tomography; INR, international normalized ratio; NSAIDs, nonsteroidal anti-inflammatory drugs; WBC, white blood cell count.

[a] Blood malignancy was included with the comorbidity of leukemia and lymphoma.

Supplementary Table 3. Characteristics of patients enrolled in predicting 1-year mortality in the derivation cohort and the validation cohort (n = 6,084)

Characteristics	Patients, no. (%)		P value[a]	Missing values
	derivation cohort (n = 4,030)	Validation cohort (n = 2,054)		
Age ≥ 70 y	2,446 (60.7)	1,232 (60.0)	0.598	0
Sex (male)	3,675 (60.4)	2,445 (60.7)	0.561	0
BMI ≤ 18.5	500 (13.1)	235 (12.1)	0.297	114 (5.6)
Current drinker	1,629 (46.7)	811 (46.1)	0.703	296 (14.4)
Current smoker	673 (18.6)	325 (18.0)	0.603	248 (12.1)
Performance status ≥ 2	455 (11.5)	225 (11.1)	0.667	20 (1.0)
Hemodynamics				
Systolic blood pressure ≤ 100 mmHg	540 (13.7)	274 (13.6)	0.968	39 (1.9)
Heart rate ≥ 100/min	791 (20.1)	401 (20.0)	0.945	47 (2.3)
Symptom				
Altered mental status	247 (6.1)	141 (6.9)	0.292	1 (0.0)
Abdominal pain	751 (18.7)	368 (17.9)	0.506	2 (0.1)
Diarrhea	463 (11.5)	233 (11.4)	0.865	4 (0.2)
Laboratory data				
Hemoglobin < 12 g/dL	2,381 (59.1)	1,175 (57.3)	0.169	2 (0.1)
WBC > 10000/mm^3	844 (21.0)	430 (20.9)	1.000	1 (0.0)
Platelet count < 150000/mm^3	653 (16.2)	289 (14.1)	**0.030**	1 (0.0)
Albumin level < 3.0 g/dL	478 (12.4)	212 (10.8)	0.072	94 (4.6)
INR ≥ 1.5	341 (9.7)	181 (10.2)	0.591	275 (13.4)
Hematocrit ≤ 35%	2,214 (55.1)	1,103 (53.9)	0.398	7 (0.3)
BUN ≥ 25 mg/dL	1,066 (26.6)	500 (24.6)	0.093	20 (1.0)

Creatinine ≥ 1.5 mg/dL	521 (13.0)	251 (12.4)	0.488	22 (1.1)
CRP ≥ 1.0 mg/dL	3,114 (77.3)	1,608 (78.3)	0.380	57 (2.8)
Home medication				
Low-dose aspirin	849 (21.1)	414 (20.2)	0.423	0
Antiplatelet drug (non-aspirin)	575 (14.3)	309 (15.0)	0.419	0
Warfarin	300 (7.4)	164 (8.0)	0.475	0
Direct oral anticoagulant	253 (6.3)	110 (5.4)	0.153	0
NSAIDs	484 (12.0)	237 (11.5)	0.615	0
Acetaminophen	94 (2.3)	51 (2.5)	0.723	0
Corticosteroid	238 (5.9)	134 (6.5)	0.337	0
Comorbidity				
Previous diverticular bleeding	601 (14.9)	340 (16.6)	0.099	1 (0.0)
Hypertension	2,217 (55.0)	1,146 (55.8)	0.567	0
Dyslipidemia	1,092 (27.1)	534 (26.0)	0.374	0
Diabetes mellitus	809 (20.1)	364 (17.7)	**0.028**	0
Diabetes complication	144 (3.6)	77 (3.7)	0.718	0
Hemiplegia	93 (2.3)	50 (2.4)	0.789	1 (0.0)
Cerebrovascular disease	568 (14.1)	270 (13.1)	0.325	0
Chronic obstructive pulmonary disease	131 (3.3)	55 (2.7)	0.238	0
Dementia	187 (4.6)	97 (4.7)	0.898	1 (0.0)
Collagen disease	186 (4.6)	97 (4.7)	0.847	0
Ischemic heart disease	642 (15.9)	331 (16.1)	0.853	0
Heart failure	338 (8.4)	186 (9.1)	0.385	1 (0.0)
Previous peptic ulcer	309 (7.7)	134 (6.5)	0.106	0
Renal failure	612 (15.2)	304 (14.8)	0.705	1 (0.0)
Peripheral arterial disease	166 (4.1)	81 (3.9)	0.784	0
Chronic hepatitis	106 (2.6)	52 (2.5)	0.865	0
Cirrhosis	97 (2.3)	41 (2.0)	0.557	0

Blood malignancy and nonmetastatic solid cancer[b]	628 (15.6)	306 (14.9)	0.499	0
Metastatic cancer	113 (2.8)	62 (3.0)	0.627	0
Diagnostic procedure				
CT	2,735 (67.9)	1,466 (71.4)	**0.005**	0
Therapeutic procedures				
Endoscopic treatment	1,323 (32.8)	673 (32.8)	0.977	0
Interventional radiology	79 (2.0)	40 (1.9)	1.000	0
Surgery	80 (2.0)	26 (1.3)	**0.049**	0
Blood transfusion during hospitalization	1,243 (30.8)	616 (30.0)	0.499	0
Final diagnosis of hematochezia				
Colonic diverticular bleeding	2,352 (58.4)	1,223 (59.5)	0.378	0
Rectal ulcer	96 (2.4)	54 (2.6)	0.600	0
Angioectasia	66 (1.6)	29 (1.4)	0.585	0
Upper gastrointestinal bleeding	74 (1.8)	30 (1.5)	0.298	0
Small intestinal bleeding	101 (2.5)	51 (2.5)	1.000	0
Cancer	111 (2.8)	36 (1.8)	**0.017**	0
Others	271 (6.7)	148 (7.2)	0.487	0
In-hospital outcomes				
Length of hospital stay ≥ 8 days	1,975 (49.0)	1,019 (49.6)	0.665	0
Rebleeding during hospitalization	598 (14.8)	284 (13.8)	0.299	0
Thromboembolism	33 (0.8)	10 (0.5)	0.194	0
Death within 1 year	163 (4.0)	82 (4.0)	0.945	0

Abbreviations: BMI, body mass index; WBC, white blood cell count; INR, international normalized ratio; BUN, blood urea nitrogen; CRP, C-reactive protein; NSAIDs, nonsteroidal anti-inflammatory drugs; CT, computed tomography.

[a] Bold values indicate P <0.05.

[b] Blood malignancy was included with the comorbidity of leukemia and lymphoma.

Supplementary Table 4. Factors and scoring algorithms included in Sengupta Score, Oakland Score, NOBLADS Score, and Charlson Comorbidity Index

Score	Variable	Categories	Points
Sengupta	Age	<30 years	−2
		30–39 years	−1
		40–49 years	0
		50–59 years	1
		60–69 years	2
		70–79 years	3
		80–90 years	4
		>90 years	5
	Dementia	Yes	5
	Chronic kidney disease	Yes	2
	Metastatic cancer	Yes	5
	Systemic anticoagulant use	Yes	1
	Chronic pulmonary disease	Yes	2
	Admission albumin	<2 g/dL	13

		2–2.9 g/dL	7
		3–3.9 g/dL	0
		≥4 g/dL	−7
Oakland	Age	40–69 years	1
		≥70 years	2
	Sex	Male	1
	Previous LGIB admission	Yes	1
	Digital rectal examination findings	Blood	1
	Heart rate	70–89/min	1
		90–119/min	2
		120–129/min	3
		130–159/min	2
	Systolic blood pressure	50–89 mmHg	5
		90–109 mmHg	4
		120–129 mmHg	3
		130–159 mmHg	2

	Hemoglobin	36–69 g/dL	22
		70–89 g/dL	17
		90–109 g/dL	13
		110–129 g/dL	8
		130–159 g/dL	5
NOBLADS	NSAIDs use		1
	No diarrhea		1
	No abdominal tenderness		1
	Systolic blood pressure	≤100 mmHg	1
	Albumin	<3.0 g/dL	1
	Charlson comorbidity index	≥2 points	1
	Syncope		1
Charlson Comorbidity Index	Myocardial infarct		1
	Congestive heart failure		1
	Peripheral vascular disease		1
	Cerebrovascular disease		1
	Dementia		1

Connective tissue disease	1
Ulcer disease	1
Mild liver disease	1
Diabetes	1
Hemiplegia	1
Moderate or severe renal disease	2
Diabetes with end-organ damage	2
Any tumor	2
Leukemia	2
Lymphoma	2
Moderate or severe liver disease	3
Metastatic solid tumor	6
AIDS	6

Abbreviations: LGIB, lower gastrointestinal bleeding; NSAIDs, nonsteroidal anti-inflammatory drugs; INR, international normalized ratio; AIDS, acquired immunodeficiency syndrome.

Supplementary Table 5. Causes of 30-day death in patients with hematochezia in the derivation cohort (n = 5,459)

**Death within 30 days**	**51**
Bleeding-related death	7 (13.7%)
Non-bleeding-related death	
Malignant disease	11 (21.6%)
Infection	11 (21.6%)
Comorbidities other than malignant disease	6 (11.8%)
Onset of acute illness	7 (13.7%)
Unknown	9 (17.6%)

Supplementary Table 6. The CACHEXIA score for 30-day and 1-year mortality in patients with acute hematochezia

Variables	30-day mortality score	1-year mortality score
Performance status ≥ 2	2	2
Liver cirrhosis	2	3
Malignancy metastasis	3	3
Albumin level < 3.0 g/dL	2	1
BUN ≥ 25 mg/dL	1	1
CRP ≥ 1.0 mg/dL	1	1
BMI < 18.5	–	1
Blood malignancy and nonmetastatic solid cancer[a]	–	2
Bleeding from malignancy	–	2
Blood transfusion during hospitalization	–	1

Abbreviations: BUN, blood urea nitrogen; CRP, C-reactive protein; BMI, body mass index.
[a] Blood malignancy was included with the comorbidity of leukemia and lymphoma.

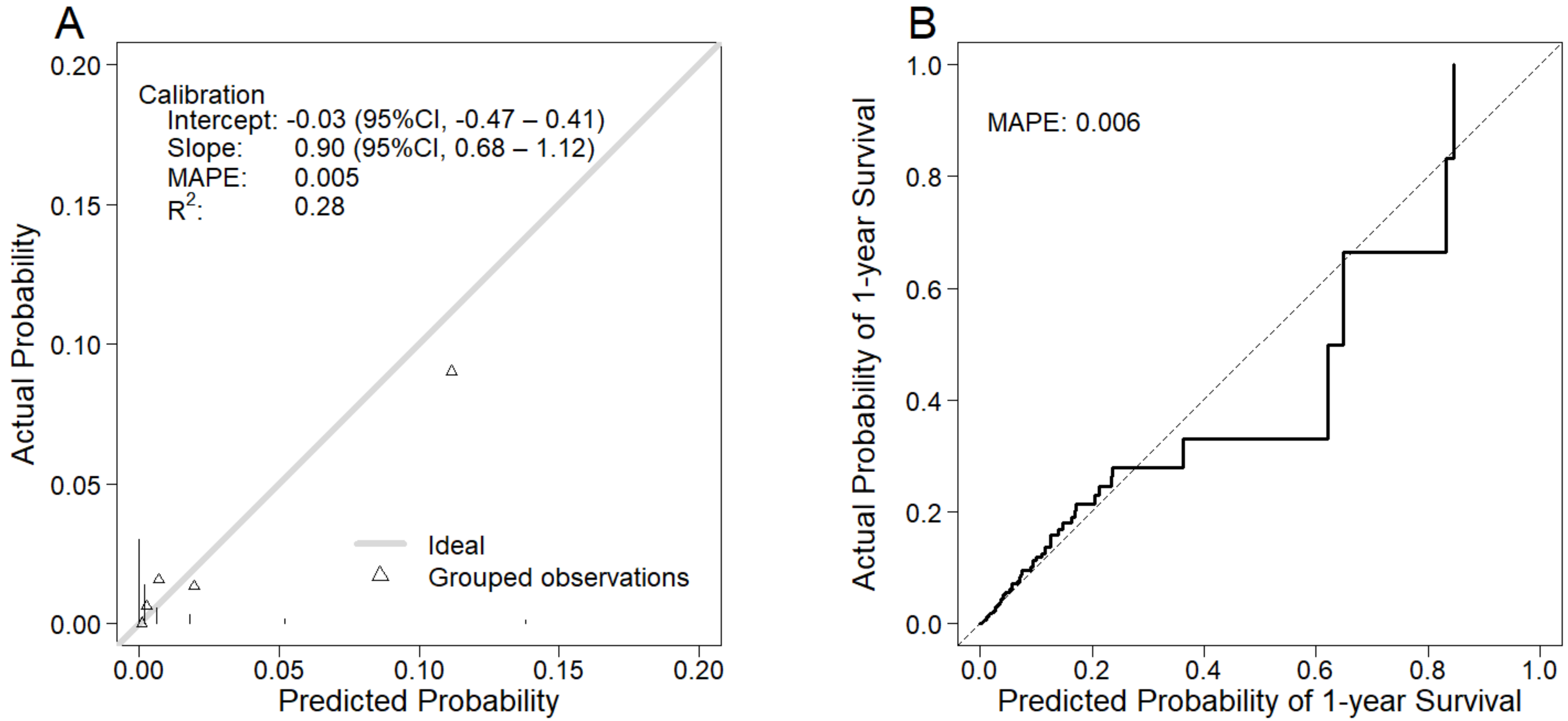

Supplementary Figure 1

Calibration plots and calculated mean absolute prediction errors. **(A)** Calibration plot for 30-day survival. **(B)** Calibration plot for 1-year survival.
Abbreviations: MAPE, mean absolute prediction error.
